# Effect of moisture content on the electromagnetic shielding ability of non-conductive textile structures

**DOI:** 10.1038/s41598-021-90516-9

**Published:** 2021-05-26

**Authors:** Sundaramoorthy Palanisamy, Veronika Tunakova, Jiri Militky, Jakub Wiener

**Affiliations:** Department of Material Engineering, Faculty of Textile, Technical University of Liberec, Liberec, 46117 Czech Republic

**Keywords:** Engineering, Materials science

## Abstract

Electromagnetically shielding textile materials, especially in professional or ordinary clothing, are used to protect an implanted pacemaker in the body. Alternatively, traditional textiles are known for their non-conductivity and transparency to an electromagnetic field. The main goal of this work was to determine whether the high moisture content (sweat) of the traditional textile structure significantly affects the resulting ability of the material to shield the electromagnetic field. Specifically, whether sufficient wetting of the traditional textile material can increase its electrical conductivity to match the electrically conductive textiles determined for shielding of the electromagnetic field. In this study, cotton and polyester knitted fabric samples were used, and two liquid medias were applied to the samples to simulate human sweating. The experiment was designed to analyse the factors that have a significant effect on the shielding effectiveness that was measured according to ASTM D4935. The following factors have a significant effect on the electromagnetic shielding effectiveness of moisturised fabric: squeezing pressure, drying time and type of liquid media. Additionally, the increase of electromagnetic shielding was up to 1 dB at 1.5 GHz frequency at the highest level of artificial sweat moisturised sample.

## Introduction

Recent technological development in the electrical and electronic industry leads to issues with electromagnetic interference. EMI, which is a radiation that interrupts the function of electronic devices and equipment^1^. EMI is the disturbance when two or more magnetic fields interact with each other. The electric field and magnetic field are always interlinked; any change in an electric field is accompanied by a change in the magnetic field and vice versa^2,3^. Many devices, such as electric motors, personal computers, laptops, electronic calculators, digital printers, internet modems, electronic typewriters, circuits, transmission lines, television, FM/AM radio, Wi-Fi routers and mobile phones emit electromagnetic (EM) waves, can result in EMI problems^4^. The EM spectrum contains an array of EM waves increasing from extremely low frequency and very low frequency, through radio frequency and microwaves to infrared (IR) light, visible light, ultraviolet (UV) light, X-rays, and gamma rays^5^. EMI is classified into two major types: man-made and naturally occurring EMI. Man-made EMI occur by electric and electronic devices such as adaptors, electrical transmitter, satellite dish and lighting. Natural EMI occurs by cosmic noise, lightning, snowstorm, rain and atmospheric noise. EMI cause data loss in communication, interruption of electronic devices, generates heat inside cells and cause of health risks, which can reduce the life of electronic devices and human^6,7^. Therefore, it is essential to shield the electronic devices against all interference sources of EM energy. Shielding is necessary to prevent devices from the negative affect by EM waves. EM shielding can be described as prevention of EM radiation transmission by a material^8^.

EM shielding materials used in medical, defence, electronic and other industries are conductive metals, wires, foams and conductive polymers^9,10^. Textile composite materials, produced using conductive polymer coating^11–13^, metal coating^14,15^, hybrid metal^5,16^, carbon nano-tubes coating^17^, and carbon yarn^18,19^ are used to protect human beings from EM radiation.

Very few studies have evaluated the moisture content on EM shielding property, and there are fewer in textile materials. The moisture content on textiles vary from material to material; for example, the moisture content (%) of linen, cotton, viscose, wool, PES and glass are 10.5, 7.5, 10, 14, 0.3 and 0%, respectively, at T = 21 ± 2 °C and a relative humidity RH = 65 ± 5%^20,21^. Another study shows that the increase in moisture content on stainless steel/ polypropylene woven fabric increased the EM shielding property. Fabric containing 20% and 100% moisture shows EM shielding effectiveness (SE) of 22 dB and 27 dB at 1.5 GHz, 24 dB and 28 dB at 2.45 GHz^22^. In this study, the EM SE of saltwater in glass and acrylic media was compared. The increase in saltwater salinity ppm from 35 to 200 increased the SE in the X-band frequency range. The multilayer saltwater material exhibited a SE of 20 dB in the X-band at a salinity of 200 ppm. The moisture content also significantly affects the EM shielding effectiveness of textile materials^23^.

A double-layer fabric was prepared using PPy coated cotton woven fabric and polyester/nylon/stainless steel hollow spindle yarn warp knitted fabric. The EM SE of the PPy woven, warp knitted and double-layer fabrics was 8, 7.5 and 14 dB, respectively, at 1.5 GHz. The simulated human sweat solution was prepared per AATCC 195 to create a double-layer fabric. However, the EM SE results of the fabric with moisture content were not reported^24^.

There are fewer studies on the effect of moisture content-related studies using EM SE. Specifically, there is no work showing the effect of the moisture content of non-conductive textile materials on EM SE. In this study, the non-conductive textile materials were treated with two different liquid mediums to test for EM SE. This study focused on the behaviour of human sweat along with the textile material against EM waves. To determine the effect of various liquid media on EM SE, the distilled water and alkaline synthetic sweat solutions were used as a liquid medium. The design of experiment (DoE) technique was used to analyse the factors significantly affecting the EM SE results.

## Mechanism of electromagnetic shielding of a textile material

EM shielding has three main mechanisms: reflection, absorption and multiple-reflection or scattering of the EM wave^25,26^. In shielding by reflection, the material must have mobile charge carriers (electrons or holes) to interact with the incoming EM waves; higher electrically conductive materials, such as silver, gold, copper, steel and carbon, have excellent reflecting property EM waves^27^. Absorption is the second most important mechanism, and it depends on the thickness of the material. The highly magnetic permeable materials mostly absorb the EM waves; for example, mu-metal, ferrite and zinc have perfect EM wave absorption properties. In conductive materials, the absorption of radiation can also occur by resistive losses, transforming the EM energy in heat by the Joule effect. The third shielding mechanism is multiple-reflection, which affects the overall shielding. If the shield is thinner than the skin depth, multiple-reflection should be considered in shielding loss, and it can be ignored in cases where the shield is thicker than the skin depth. Conductive textile materials are the best example of the multiple-reflection of EM waves because of their structure.

The plane wave shielding theory developed by Schelkunoff and Schultz et al.^28^ defines the SE as shown in Eq. (1):

1 $$SE = A + R + B$$

where B is a term that considers the loss caused by multiple reflections inside the shield, *R* is the reflection loss and *A* is the absorption loss. The SE is represented in unit decibel [dB], which means the power or intensity ratio.

Operation of the EM shield can be characterised by the shielding attenuation coefficient (ES) (dimensionless), which defines a ratio between EM field density in a specific place of shielded space *P*_t_ and incident EM field density *P*_i_ (Eq. 2).

2 $$ES = \frac{{P_{t} }}{{P_{i} }} \left[ - \right]$$

The logarithmic size of this coefficient is called electromagnetic shielding effectiveness (EM SE), which is used more frequently in the *SE* calculation (Eq. 3).

3 $$SE = 10log\frac{{P_{t} }}{{P_{i} }} = 20log\frac{{E_{t} }}{{E_{i} }} = 20log\frac{{H_{t} }}{{H_{i} }} \left[ {dB} \right]$$

where *H*_*t*_, *E*_*t*_ and *P*_*t*_ are the electric field strength, magnetic field strength and EM field density values measured in the presence of the textile material. *H*_*i*_, *E*_*i*_ and *P*_*i*_ are the same values measured without the textile material.

### Electromagnetic wave transmission mechanics

In Eq. (4), the speed of EM waves (*ν*) is defined, indicating two main parameters, *µ* (permeability) and *ε* (permittivity)^2^. This equation is unclear on whether moisture content influences the relative permeability of the textile media. In any media, the EM waves enter the primary mechanism of reflection and secondary mechanism of absorption; transmission then occurs. The energy distribution coefficient *γ* is defined in Eq. (5). The relative permeability of the textile substrate is assumed to be one; *γ* is shown in Eq. (6). There is insufficient reason to consider the relative permeability influence on EM transmission.

4 $$\nu = \frac{1}{{\sqrt {{\upmu }\varepsilon } }}$$

5 $$\gamma = \frac{{\left( {\begin{array}{*{20}c} {\sqrt {\varepsilon_{2} \mu_{2} } } & { - \sqrt {\varepsilon_{1} \mu_{1} } } \\ \end{array} } \right)^{2} }}{{\left( {\begin{array}{*{20}c} {\sqrt {\varepsilon_{2} \mu_{2} } } & { + \sqrt {\varepsilon_{1} \mu_{1} } } \\ \end{array} } \right)^{2} }}$$

6 $$\gamma = \frac{{\left( {\begin{array}{*{20}c} {\sqrt {\varepsilon_{2} } } & { - \sqrt {\varepsilon_{1} } } \\ \end{array} } \right)^{2} }}{{\left( {\begin{array}{*{20}c} {\sqrt {\varepsilon_{2} } } & { + \sqrt {\varepsilon_{1} } } \\ \end{array} } \right)^{2} }}$$

### Mechanics of moisture content in the material influencing relative permeability

All non-magnetic materials have relative permeability, and the non-conductive textile materials are also assumed to be one^25^. Relative permittivity *ε*_*0*_ is also expressed as a relative complex permittivity *ε*_*r*_, as shown in Eq. (7). *ε*_*r*_ is a dimensionless complex number and the representation of real part ${\upvarepsilon }_{r}^{^{\prime}}$ is the storage coefficient of the media on the EM waves, which indicates that the EM wave propagation speed in the media is affected. The representation of imaginary part ${\upvarepsilon }_{r}^{^{\prime\prime}}$ is the loss capacity of the media to the EM wave. A penetration depth is defined as the distance EM waves penetrate the media, as shown in Eq. (8). The reduction coefficient *α* and penetration depth *dp* directly influence the relative permittivity of the media. For textile specimens, the proportion of moisture inside is influenced by the reduction level of EM wave penetration into textile media.

7 $$\varepsilon_{r} = \varepsilon_{r}^{^{\prime}} - j\varepsilon_{r}^{^{\prime\prime}} = \frac{\varepsilon }{{\varepsilon_{0} }} - j \frac{\delta }{{\omega \varepsilon_{0} }} = \varepsilon_{r}^{^{\prime}} \left( {1 - j \tan \sigma } \right)$$

8 $$d_{p} { } = \frac{{{ }1{ }}}{{\upalpha }}{ } = { }\frac{{1{ }}}{{\uppi }}{ }\frac{{{\upvarepsilon }_{r}^{^{\prime}} }}{{{\upvarepsilon }_{r}^{^{\prime\prime}} f}}{ }\sqrt {\frac{{1{ }}}{{{\upvarepsilon }_{r}^{^{\prime}} { } \times {\upvarepsilon }_{0} \times {\upmu }_{0} }}}$$

*ε*_*0*_ is the relative permittivity of a vacuum (8.854 × 10^−12^ F/m), ε is the relative permittivity of the media (F/m), *σ* is the conductivity of media (S/m), *ω* is the angular frequency of the EM wave, ${\upvarepsilon }_{r}^{^{\prime}}$ and ${\upvarepsilon }_{r}^{^{\prime\prime}}$ are the real and imaginary parts of relative permittivity, respectively, j = √ − 1, tan δ = ${\upvarepsilon }_{r}^{^{\prime\prime}}$ /${\upvarepsilon }_{r}^{^{\prime}}$, *µ*_*0*_ is the magnetic permeability of vacuum (4π × 10^−7^ H/m) and *f* is the frequency of an EM wave.

### Influence of relative permittivity on the electromagnetic waves response

The transmission of EM waves inside textile media obeys the Maxwell equations^29^, which are shown in Eqs. (9)–(12). Equations (13) and (14) define the *µ* (permeability) and *ε* (permittivity), respectively^30^. By combining these equations, the magnetic induction intensity is influenced by *µ* and *ε*, which indicates the reduction level of the EM wave response:

9 $$\nabla { } \times {\text{ E }} = { } - { }\frac{{\partial {\text{B }}}}{{\partial {\text{t}}}}$$

10 $$\nabla { } \times {\text{ H}} = {\text{ J}} + { }\frac{{\partial {\text{D }}}}{{\partial {\text{t}}}}$$

11 $$\nabla { } \times {\text{ B }} = { }0$$

12 $$\nabla \times D = \rho$$

13 $$D = \varepsilon E$$

14 $$B = \mu H$$

where *ρ* is the charge density (C/m^3^), *J* is the current density (A/m^2^), *E* is the intensity of the EM wave (V/m), *D* is the electric displacement (C/m^2^), *B* is the magnetic induction intensity or magnetic flux density (T), *H* is the magnetic field intensity (A/m), *ε* is the permittivity and *µ* is the permeability of the media. For instance, the permittivity of an EM wave in a vacuum is 8.85 × 10^−12^ F/m and the permeability in a vacuum is 4π × 10^−7^ H/m.

Cotton and polyester fabric samples were used for analysis and their specifications are shown in Table 1. The different samples, bleached cotton fabric and detergent treated polyester, were chosen based on market availability and ready to wear. The two different liquid mediums, distilled water and artificial sweat solution (alkaline solution) were used to treat fabric samples. The artificial human sweat medium was prepared per ISO 105-E04. The fabric samples were soaked in prepared liquid mediums for 1 h, and a padding mangle was introduced to squeeze the fabric samples at 0, 1.25 and 2.5 bar pressures. The samples were weighed before and after squeezing. The squeezed samples were dried at room temperature (21 ± 2 °C at 65 ± 5% RH) for three different drying times (0, 60 and 120 min), then tested for EM SE and the moisture content (MC) % was calculated.

**Table 1:** Knitted fabric parameters.

Parameters	Results	
Material	Cotton	Polyester
Fabric description	Single Jersey	Interlock
Course per inch (ends/inch)	42	43
Wales per inch (ends/inch)	33	32
Loop length (cm)	0.287	0.289
Yarn count (Ne)	30.22	30.47
Mass per square meter	155	114
Fabric thickness (mm)	0.35	0.22

## Experimental results

The three liquid mediums were distilled water, acidic sweat and alkaline sweat solutions, and their measured electrical conductivity was 0.0016, 9.29 and 10.29 mS/cm, respectively. The sweat solutions had 50 times higher electrical conductivity than distilled water.

The cotton and polyester fabric samples were treated with distilled water and artificial sweat solutions for 1 h. The sample was then squeezed using three different pressures and three different drying times were used to determine the MC% for the tested samples. The calculated values of the MC% is shown in Table 2.

**Table 2:** Moisture content (MC %) of the cotton and polyester fabric samples.

Material	Squeezing pressure (bar)	MC %								
		Distilled water			Alkaline sweat			Acidic sweat		
		Drying time (minute)								
		0	60	120	0	60	120	0	60	120
Cotton	0	205	138	91	217	167	111	206	153	101
	1.25	127	78	44	130	86	52	126	73	35
	2.5	110	61	25	112	58	24	110	62	18
Polyester	0	249	153	86	251	159	96	244	168	85
	1.25	183	109	51	189	119	62	185	125	61
	2.5	156	80	22	164	80	25	154	86	20

## Electromagnetic shielding results of moist fabrics

**Figure 1:**
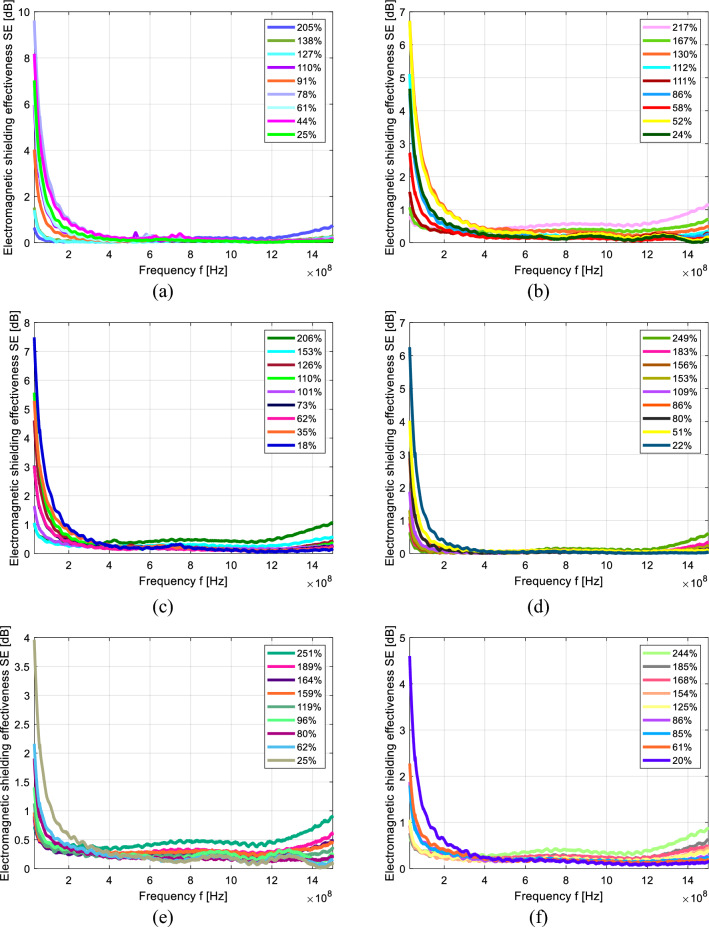
EM SE versus frequency (30 MHz to 1.5 GHz) of cotton fabric treated in (**a**) distilled water, (**b**) alkaline sweat and (**c**) acidic sweat. Polyester fabric treated in (**d**) distilled water, (**e**) alkaline sweat and (**f**) acidic sweat with different MC%.

Figure 1 shows the EM SE versus frequency graph of the liquid media treated cotton and polyester knitted samples. Figure 1a shows the SE versus frequency graph of the cotton (COT) fabric sample treated with distilled water. The SE at 1.5 GHz decreases with decreasing MC in the cotton samples. At 1.5 GHz, the MC% of 205, 110 and 25 has a SE of 0.7, 0.2 and 0.1 dB, respectively. At 30 MHz, the SE increases with in MC of the cotton fabric. At 30 MHz, the MC% of 25, 110 and 205 has an SE of 7, 6 and 0.6 dB, respectively. The alkaline and acidic sweat-treated COT samples are shown in Fig. 1b,c. Both the sweat-treated samples have higher SE values at 30 MHz compared with 1.5 GHz. The acidic treated samples have greater SE at 30 MHz; however, at 1.5 GHz, both the acidic and alkaline treated samples exhibited the same SE at the same MC. The alkaline treated samples containing MC% of 217, 111 and 24 has a SE of 1.1, 0.3 and 0.1 dB, respectively, at 1.5 GHz. The acidic treated samples containing MC% of 206, 110 and 18 have a SE of 1.1, 0.3 and 0.2 dB, respectively, at 1.5 GHz.

Figure 1d shows the SE versus frequency graph of the polyester (PES) fabric sample treated with distilled water. The SE at 1.5 GHz decreases with decreasing MC in the polyester samples. At 1.5 GHz, the MC% of 249, 109 and 22 samples have a SE of 0.6, 0.1 and 0 dB, respectively. At 30 MHz, the SE increases with decreasing MC of the PES fabric. At 30 MHz, the MC% of 22, 109 and 249 has a SE of 6.2, 1.9 and 0.6 dB, respectively. The alkaline and acidic sweat-treated PES samples are shown in Fig. 1(e & f). Both the sweat-treated samples have higher SE values at 30 MHz compared with 1.5 GHz. The acidic treated samples have higher SE at 30 MHz; however, at 1.5 GHz, both the acidic and alkaline treated samples exhibit the same SE at the same MC. The alkaline treated samples containing MC% of 251, 119 and 25 has a SE of 0.9, 0.3 and 0.1 dB, respectively, at 1.5 GHz. The acidic treated samples containing MC% of 244, 125, and 20 have a SE of 0.9, 0.4 and 0.1 dB, respectively, at 1.5 GHz.

The distilled water treated samples have higher SE at 30 MHz compared with sweat-treated samples. At a higher frequency of 1.5 GHz, the sweat-treated samples are slightly greater SE than the distilled water treated samples.

The PES has a faster evaporation rate of the liquid medium at room temperature (RT) than COT (Table 2) because the PES is hydrophobic and COT is hydrophilic. PES initially holds more moisture than COT because PES holds more fluid in-between yarns. The hydrophobic nature of PES tends to adsorb fluid on its surfaces, which leads to more moisture. Additionally, the PES and COT MC at RT was 0.3% and 7.5%, respectively. The sweat solution treated PES and COT samples have slightly higher SE (at all frequency range) then distilled treated samples because the sweat solution contains 5 gpl of Na^+^ Cl^−^ and it acts as a semi-conductor, which is why the sweat solution has a higher SE than distilled water at a higher frequency range.

## Design of experiment analysis

In DoE, the EM SE is inhomogeneous because the SE is affected by various parameters, including the liquid medium (A), material (B) and MC (C). A represents the two liquid mediums: acidic and alkaline sweat solutions; B represents the two materials: polyester and cotton knitted fabrics samples, and C represents the three different ranges of percent MC of the samples. For investigating the influence of MC on SE, a full factorial design contains two levels of A and B factors and three levels of C factor with three replicates. The three levels of factor C are ‘1’ (MC 200%), ‘0’ (MC 110%) and ‘ − 1’ (MC 25%). The response is the SE value in dB at 1.5 GHz. The factors and their levels are shown in Table 3.

**Table 3:** Factors and their levels.

Level	A Liquid medium	B Material	C Moisture content [%]
Low	Acidic water	Polyester	− 1
Medium	–	–	0
High	Alkaline sweat	Cotton	1

There were forty-two runs in the design, and the results were tested at random to improve the experimental accuracy. Table 4 shows the mean SE values (from three replicates) for each combination of factors.

**Table 4:** The factors and their mean shielding effectiveness at 1.5 GHz.

A (Liquid medium)	B (Material)	C (Moisture content)	Response SE [dB] @ 1.5 GHz
Alkaline	Polyester	− 1	0.2
Alkaline	Polyester	1	0.8
Alkaline	Cotton	− 1	0.1
Alkaline	Cotton	0	0.3
Acidic	Polyester	0	0.4
Acidic	Polyester	1	0.9
Acidic	Cotton	− 1	0.2
Acidic	Cotton	1	0.8

Table 5 shows the results of the factorial analysis. The p-value is the probability (within a 95% confidence level) that the selected factors have a statistically significant impact on the response (SE of materials). The impact is significant when its p-value ≤ 0.05. In this experiment, the main factors, liquid medium, A, and MC, C, significantly affect SE. The cross effects have no significant effect on SE.

**Table 5:** Factorial fit: SE versus A, B, and C.

Term	Coef	SE Coef	T-Value	P-Value	VIF
Constant	0.3535	0.0462	7.65	0.000	
A	− 0.0398	0.0192	− 2.07	0.046	1.06
B	− 0.0132	0.0192	− 0.68	0.498	1.06
C	0.3257	0.0229	14.25	0.000	1.07
CxC	0.1181	0.0653	1.81	0.080	2.50
AxB	0.0082	0.0302	0.27	0.789	2.57
AxC	− 0.0038	0.0247	− 0.15	0.878	1.21

**Model summary**	S	R-sq	R-sq (adj.)	R-sq (pred)	
	0.12	88.37%	85.55%	81.49%	

‘p-value’: a 5% significance level; *Coef.* coefficient; *adj.* adjusted, *pred.* predicted.

Furthermore, the C factors have more significant effect on SE than A. The variance inflation factor is also shown in Table 5. The model is reliable when the variance inflation factor (VIF) ≤ 10.

Equation 15 represents the regression model. From the analysis of variance (ANOVA), the R-sq (adj) value of 85.55% indicates that the proposed regression model can explain 88.37% of the variation of load hysteresis. The predictability of the regression model is 81.49%.

15 $$\begin{aligned} SE \left( {dB} \right) & = 0.3535 - 0.0398A - 0.0132B + 0.3257C \\ & \quad + 0.1181CxC + 0.0082AxB - 0.0038AxC \\ \end{aligned}$$

**Figure 2:**
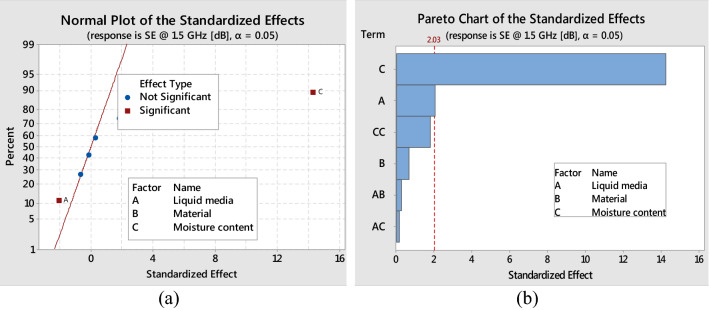
Standardised effects on SE (dB) at 1.5 GHz: (**a**) normal plots and (**b**) Pareto chart.

The normal probability plot of the standardised effects in Fig. 2a indicates the relative magnitude and statistical significance of the main and interaction effects. The main factors (A, C) had a significant effect on fabric SE at 1.5 GHz. Furthermore, any effect beyond the reference line is regarded as significantly large. Figure 2b shows the Pareto chart, which also indicates significant and insignificant factors by separation with the red line marked at 2.04. The Pareto chart of the standardised effects shows that factor C had a more significant effect than factor-A.

To check the adequacy of the regression models, the residuals were examined for the acceptability of three basic assumptions of normality, constant variance and independence. The residuals are the difference between the calculated value and observed value.

**Figure 3:**
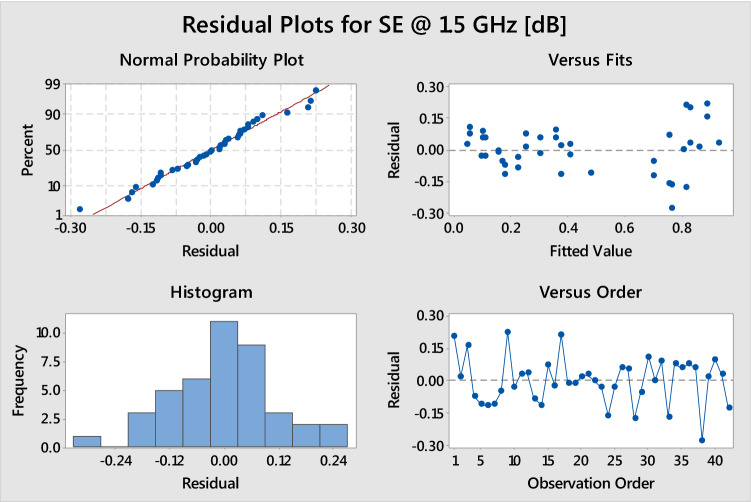
Residual plots for SE for process development.

The residual plots in Fig. 3 are essential to validate the regression model. The normal probability, residual versus fit, histogram and residual versus order confirm the adequacy of the regression model. The normal probability plot shows that the points are close to a straight line, which confirms that the normal distribution and the proposed regression model are reasonable. The histogram confirms the distribution frequency of residuals is normal. The residual versus fit is to verify if the residuals are distributed randomly; here, the points are random and reasonable for the normality and constant variance of the data. The residual versus order is helpful to verify the assumption. As shown in Fig. 3, the observed orders are independent of one another; hence, the independence assumption is accepted.

**Figure 4:**
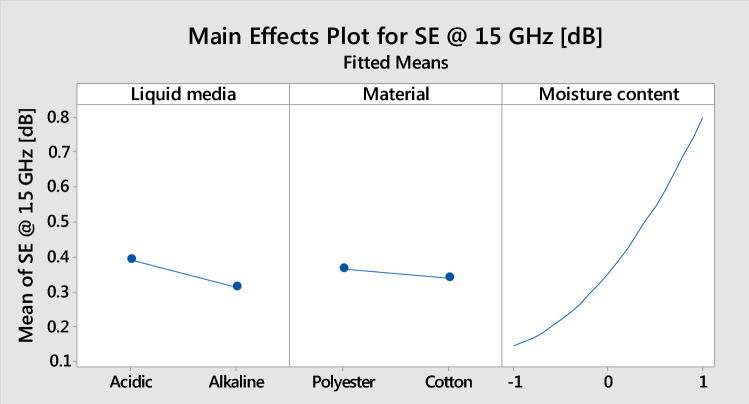
Main factors plot for SE at 1.5 GHz.

The main factors and their interaction effects are shown in Fig. 4. Regarding the main factor plot for SE, factors A and C plays the most crucial role in affecting the SE. The cotton fabric treated with alkaline media containing moisture less than 62% has a lower SE value. The higher MC of acidic media treated sample led to higher SE. For liquid medium, the sweat solutions have a slightly significant effect on SE. The materials had no effect on SE, and the graph was nearly parallel. The main effect plot concludes that the higher the MC, the higher SE.

**Figure 5:**
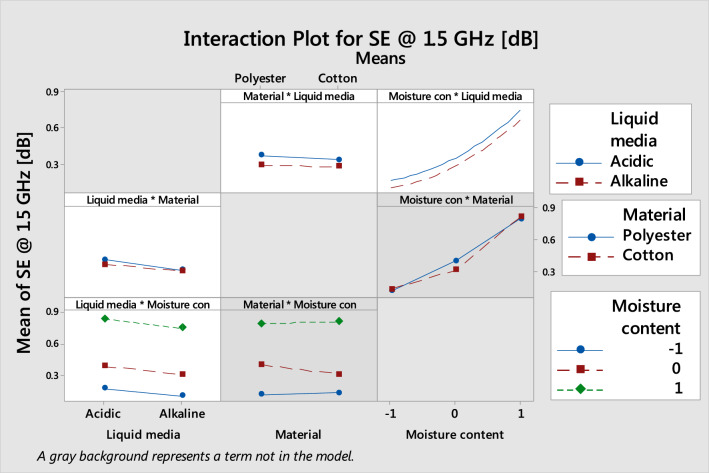
Interaction plots for SE at 1.5 GHz.

According to the interaction plot for SE shown in Fig. 5, the SE of the main factors shows that they have cross effects, verifying the proposed regression Eq. (4). The interaction plot is helpful to predict the response by varying the level of the factors. Additionally, it helps to choose the combination of factors that require response value. The parallel line indicates no interaction; the other trend line indicates interaction. The liquid medium has a smaller effect on the SE value. For sweat mediums, the SE values slightly trended with the material and MC. Both the cotton and polyester materials have slightly higher SE in acidic medium. The increase in MC increases the SE, irrespective of the liquid medium. The material does not affect liquid media and MC, and its line is almost parallel. The MC has a significant influence on the SE. Both the cotton and polyester materials SE increased from 0.1 dB to 0.8 dB with the increased MC. Additionally, both the acidic and alkaline media SE value increased from 0.1 dB to 0.9 dB with increasing MC. During the material survey, the SE was nearly parallel (no interaction) in the material.

## Conclusion

Polyester and cotton weft-knitted samples were taken for analysis of the MC effect on SE. Acidic sweat, alkaline sweat and distilled water were used as a liquid medium for MC analysis. In this experiment, the alkaline and acidic sweat solutions are used to simulate human sweating; however, human sweat was mostly in acidic pH. EM shielding versus frequency results shows that the cotton treated with sweat has the highest shielding, e.g., 1.1 dB at 1.5 GHz. At all liquid media, the lowest MC in the material has higher SE at 30 MHz, and the higher MC in the material has higher SE at 1.5 GHz. The rate of drying of the polyester is faster than cotton because of its hydrophilic nature.

The alkaline and acidic sweat solutions were used in a DoE. The main factors of liquid medium and MC, have a statistically significant effect on EM SE at 1.5 GHz. The cross-effect (moisture content by moisture content) have a significant effect on SE. The acidic sweat solution treated cotton fabric at a higher MC level has the highest SE (0.9 dB at 1.5 GHz) with a MC of 206%. Therefore, the highest MC in the fabric sample would exhibit higher EM SE. Additionally, human sweat has a significant effect on EM radiation shielding at lower frequencies. However, the SE of moisturised non-conductive textile materials was very low compared with electrically conductive textile materials. A limitation of this study is that only the variability of SE within the material was considered.

## Methods

### Electromagnetic shielding effectiveness evaluation

The SE of the sample set was measured according to ASTM D4935-18 for the planar materials using a plane wave, the far-field EM wave at the *RT* = 21 ± 2 °C and the relative humidity *RH* = 54 ± 5%. The SE of the samples was measured over the frequency range of 30 MHz to 1.5 GHz. The set-up consisted of a sample holder with its input and output connected to the network analyser. A SE test fixture (Electro-Metrics, Inc., USA, Model EM-2107A) was used to hold the sample. The design and dimension of the sample holder follow the ASTM method mentioned above. A vector analyser Rohde & Schwarz ZN3 was used to generate and receive the electromagnetic signals. The standard determines the SE of the fabric using the insertion-loss method, and it has an error of ± 3 dB. A reference measurement for the empty cell was required for the shielding effectiveness assessment. A “through” calibration with a reference sample was made first. A load measurement was subsequently performed on a solid disc shape sample. The reference and load specimens must be of the same material and thickness. Both the reference and load samples geometries were performed according to ASTM D 4935-18. The measurements were performed at three different places of the textile samples because of the subsequent statistical analysis^31^.

### Fabric properties

The areal density of the fabric (*w*) [g/m^2^] was measured with the sample size of 100 cm^2^, per ASTM D 3776 standard. The thickness of the fabric (*t*) was measured using the thickness gauge [mm], per the standard ASTM D1777.

### Moisture content

The MC percent of the sample is tested per ASTM D2495-07. The percentage of moisture present in the fabric was calculated using the below formula (Eq. 16).

16 $$Moisture content, MC \left( \% \right) = \frac{W}{{\left( {W + D} \right)}} \times 100$$

where *W* is the weight of water present in the fabric (original weight –oven dry weight) and *D* is the oven dry weight of the fabric. The oven-dry weight is the constant weight obtained by drying fabric at a specific temperature of 105 ± 3 °C.

### Electrical conductivity of the liquid medium

The electrical conductivity of the liquid medium was tested per ASTM D1125-14.

### Experimental design

The DoE was used to investigate the influence of process parameters, such as the type of liquid medium, squeezing pressure, drying time and type of materials, on the EM SE. The DoE tests the predictions using screening design or Plackett–Burman design. Plackett–Burman design helps determine the result with a very economical design with the number of runs in multiples of four and provides a very effective screening design with an interest in main effects. Minitab® 18.0 Software was used for the experimental design and statistical analysis of the experimental results. The statistical significance was estimated by two-way ANOVA and a p-value < 0.05 was used as the criterion for significance.
